# Diverse Phenotypes and Specific Transcription Patterns in Twenty Mouse Lines with Ablated LincRNAs

**DOI:** 10.1371/journal.pone.0125522

**Published:** 2015-04-24

**Authors:** Ka-Man Venus Lai, Guochun Gong, Amanda Atanasio, José Rojas, Joseph Quispe, Julita Posca, Derek White, Mei Huang, Daria Fedorova, Craig Grant, Lawrence Miloscio, Gustavo Droguett, William T. Poueymirou, Wojtek Auerbach, George D. Yancopoulos, David Frendewey, John Rinn, David M. Valenzuela

**Affiliations:** 1 VelociGene, Regeneron Pharmaceuticals, Inc., Tarrytown, New York, United States of America; 2 Department of Stem Cell and Regenerative Biology, Harvard University, Cambridge, Massachusetts, United States of America; 3 Broad Institute of MIT and Harvard, Cambridge, Massachusetts, United States of America; Harbin Institute of Technology, CHINA

## Abstract

In a survey of 20 knockout mouse lines designed to examine the biological functions of large intergenic non-coding RNAs (lincRNAs), we have found a variety of phenotypes, ranging from perinatal lethality to defects associated with premature aging and morphological and functional abnormalities in the lungs, skeleton, and muscle. Each mutant allele carried a *lacZ* reporter whose expression profile highlighted a wide spectrum of spatiotemporal and tissue-specific transcription patterns in embryos and adults that informed our phenotypic analyses and will serve as a guide for future investigations of these genes. Our study shows that lincRNAs are a new class of encoded molecules that, like proteins, serve essential and important functional roles in embryonic development, physiology, and homeostasis of a broad array of tissues and organs in mammals.

## Introduction

It has recently become clear that an in-depth understanding of the relationship between genotype and phenotype in mammals requires that we expand our investigations beyond the protein-coding genes to include the non-coding portion of the genome [1]. Large-scale whole genome expression studies in mammalian cells have revealed that approximately three-quarters of the genome is capable of being expressed as RNA [2–4], and most of the transcripts do not code for proteins. Among the non-coding transcripts is a diverse class known as long non-coding RNAs (lncRNAs). Representing approximately 15,000 transcripts from nearly 10,000 genomic loci in human cells [5], lncRNAs and a subclass known as large intergenic non-coding RNAs (lincRNAs) [6,7] resemble protein-coding mRNAs in structure, synthesis, and the chromatin character of their genes. Whether or not this structural similarity extends to a functional diversity that matches that of proteins remains an open question.

Since the creation of the first knockout strain nearly twenty-five years ago, the mouse has become the premier system for the study of mammalian gene function [8–10]. With few exceptions, the application of knockout mouse technology in individual gene studies as well as large-scale international projects (http://www.knockoutmouse.org) has focused on protein-coding genes, but the recent efforts to create global knockout mouse resources for microRNAs [11] (http://mcmanuslab.ucsf.edu/microrna_knockout) demonstrate the value of applying the technology to non-coding RNAs. There have been a few functional studies of individual lncRNAs by gene disruption in mice, but about half have focused on well-studied lncRNAs involved in two related biological phenomenon: X chromosome inactivation [12,13] and somatic chromosome imprinting [14–17]. Recently, disruption of the mouse *Fendrr* lncRNA resulted in embryonic lethality associated with defects in heart and body wall development [18]. However, deletion or insertion mutations in the lncRNA-encoding Gt(ROSA)26Sor [19] or Malat1 [20] genes produced no discernable phenotypes. The emerging understanding of the structure, expression, and function of the lncRNA genes presents a new opportunity to employ mouse molecular genetics to reveal the biological functions associated with this new class of genes.

Applying knockout mouse technology to lncRNAs does, however, present some technical challenges. Most proteins have elements or domains that are known or at least predicted to be of functional relevance. Deleting the coding sequences for these essential parts is often sufficient to create a null allele. Likewise, conditional alleles can be designed that isolate the critical exon or exons for later deletion by the action of a tissue specific recombinase. Because structure-function relationships have not yet been established for all but a few lncRNAs and there is no open reading frame as a guide, the knockout strategies available to protein-coding genes may not be applicable to the genomic loci that encode lncRNAs. Although the annotation of lncRNA genes has improved [5], the precise boundaries of some genes may still remain ambiguous, which can complicate knockout allele design. A powerful tool applied to knockout mice for protein-coding genes is the replacement of the target gene with a reporter, such as the coding sequence for ß-galactosidase or a fluorescent protein, whose expression is controlled by the target gene’s promoter, thereby reporting the spatial and temporal pattern of its expression in the mouse. Reporter gene replacement has been applied successfully to non-coding RNAs such as the well-studied Gt(ROSA)26Sor locus [19], which encodes a lncRNA, and the gene for the small non-coding RNA miR-155 [21], but rules for creating such alleles for lncRNAs may need to be developed. Despite these qualifications, with thousands of lncRNAs identified, the time is ripe to apply the power of knockout mouse technology to this new class of genes. In addition, the lncRNAs may likely function primarily in higher-level phenomenon such as development and aging that require whole-animal models for investigation. To this end, we describe here a unified genetic approach to elucidate the functions of twenty lincRNAs by the creation of knockout mouse lines, each carrying a gene-ablating deletion allele with a ß-galactosidase reporter replacement.

## Materials and Methods

### Generation of lincRNA Knockout Mice

We employed the VelociGene method as described previously, which enables the rapid and high-throughput generation of custom gene mutations in mice [22]. Briefly, BacVec large targeting vectors were generated using BAC clones from the mouse bMQ (129S7/SvEv Brd-Hprt b-m2) or RP23 BAC library [23] and introduced into F1 hybrid (129S6SvEvTac/C57BL6NTac) ES cells followed by culturing in selection medium containing G418. Drug-resistant colonies were picked 10 days after electroporation and screened for correct targeting by the loss-of-allele assay [22,24]. We used the VelociMouse method [25–27], in which targeted ES cells were injected into uncompacted 8-cell stage Swiss Webster embryos, to produce healthy fully ES cell-derived F0 generation mice carrying the lincRNA knockout mutations. The genomic coordinates of the targeted deletions for each lincRNA are given in Table 1.

**Table 1:** LincRNA Knockout Deletion Alleles.

	Deletion Properties		
lincRNA Gene	Deletion start exon^a^	Size (kb)	Genomic Coordinates^b^
*Celrr*	E2	50	Ch1: 121087772–121137464
*Crnde*	E2	25	Ch8: 92325913–92350749
*Eldr*	E1	17	Ch11: 16934419–16951083
*Fendrr*	E2	19	Ch8: 121054882–121074065
*Haglr*	E1	12	Ch2: 74750433–74762886
*Halr1*	E2	8.6	Ch6: 52106776–52115377
*Hotair*	E1	2.3	Ch15: 102945399–102947720
	E2	0.43	Ch15: 102945399–102945826
*Hottip*	E1	4.8	Ch6: 52262834–52267603
	E2	2.2	Ch6: 52265374–52267603
*Hoxa11os*	E3	3.5	Ch6: 52246320–52249795
	E4	3.1	Ch6: 52246643–52249795
	E5	0.70	Ch6: 52249094–52249795
*Kantr*	E1	29	ChX: 152298544–152327475
*Lincenc1*	E2	26	Ch13: 97455710–97482249
*Lincpint*	E2	32	Ch6: 31166026–31197846
*Lincppara*	E1	29	Ch15: 85671665–85701064
*Mannr*	E1	32	Ch3: 29891188–29923147
*Pantr1*	E1	47	Ch1: 42648175–42694815
*Pantr2*	E1	6.5	Ch1: 42707143–42713698
*Peril*	E1	14	Ch3: 34767849–34782292
*Ptgs2os2*	E1	5.9	Ch1: 150159024–150164899
*Trp53cor1*	E1	22	Ch17: 29057474–29079078
	E2	2.9	Ch17: 29057474–29060353
*Tug1*	E1	9.0	Ch11: 3639794–3648758
	E2	5.7	Ch11: 3639794–3645518

^a^LincRNA genes often have multiple annotated transcripts. All deletions end at the last annotated exon.

^b^GRCm38 (GCA 000001635.3).

### *LacZ* Expression Profiling

Male VelociMice were used directly for *lacZ* expression profiling or mated with C57BL/6NTac females to produce embryos or adults for *lacZ* analysis. For whole-mount staining, E9.5 through E14.5 embryos were collected, washed in PBS and incubated for 15 to 60 minutes in fresh 0.2% glutaraldehyde solution. Embryo yolk sacs were taken for genotyping. After fixation, embryos were washed and incubated in X-gal (1 mg/mL) staining solution at 37°C for 1 to 24 hours. After staining, tissues were rinsed in wash buffer, post-fixed in 4% paraformaldehyde, and incubated in 70% ethanol for at least 24 hours. E9.5-E11.5 embryos were photographed immediately, while E12.5 embryos and older were cleared in a series of solutions containing increasing glycerol and decreasing 1% KOH. Photographs were taken on a Nikon SMZ1500 stereomicroscope using a Nikon DS-Ri1 digital camera. Lungs from *Fendrr* E13.5 embryos were dissected for photography after embryo clearing.

For studies with adult mice, 6 to 8-week old mice were deeply anesthetized via Ketamine/Xylazine (120/5 mg/kg) IP injection and fixed by cardiac perfusion using a 0.2% glutaraldehyde, 4% paraformaldehyde solution. Brain, ribcage, heart, lung, liver, spleen, stomach, kidney, intestine, urogenital, muscle, and hind limb tissues were dissected, rinsed in PBS and post-fixed for 30 minutes in a 0.2% glutaraldehyde, 4% paraformaldehyde solution. Tissues were then washed and incubated in X-gal (1 mg/mL) staining solution for 1 to 24 hours at 37°C. After staining, tissues were rinsed in wash buffer, post-fixed in 4% paraformaldehyde, cleared in a series of 50%, 70% and 100% glycerol and photographed.

### Animal Care and Experimental Procedures

Phenotypic studies of N2F1 mice began at 6–8 weeks of age. For timed matings, we assigned the morning of identification of vaginal plugs as embryonic day 0.5 (E0.5). LincRNA homozygous knockouts and WT littermates were observed from birth for various developmental milestones (runting, breathing, facial and limb abnormalities, skin color, posture, righting and eye opening) until about 6−8 weeks of age, when they were housed 2–5 per cage in 12 hours of light per day at 20–23°C, and 40–60% humidity for study. Mice were housed in 95.6 x 309.1 x 133.4 mm cages (Thoren, Small Mouse Cage #1) with cob bedding (The Andersons Lab Bedding, Cat# 8B) and a cotton nestlet for enrichment (Ancare, Cat #28CNEST). In housing the mice were monitored twice daily for health status and had access to normal chow (LabDiet, Cat#0007688) and water *ad libitum*. All animal procedures were carried out in strict accordance with the recommendations in the Guide for the Care and Use of Laboratory Animals of the National Institutes of Health. The protocol was approved by the Regeneron Pharmaceuticals Institutional Animal Care and Use Committee (IACUC), and all efforts were made to minimize suffering. This report adheres to the ARRIVE Guidelines for reporting Animal Research as shown with a complete ARRIVE guidelines checklist included in S1 ARRIVE Guidelines Checklist.

### RNA Expression Analysis

Total RNA was isolated from tissues of four mice from each group using RNeasy Plus Kits (Qiagen), and real-time PCR measurements were performed as described [22]. Expression level were quantified using probe set for mouse *Lincpint* RNA, (ABO41803), the assay probe spans an exon junction (Life Technologies, Assay Mm00519983_m1), and for GFP (GFP forward, 5’-CTACCCCGACCACATGAAGC; GFP reverse, 5’–TGCGCTCCTGGACGTAGC).

### MicroCT Analysis

3D skeletal imaging was visualized using the Quantum FX microCT Pre-clinical In-Vivo Imaging System (Perkin Elmer). Mice were anesthetized using oxygen/isofluorane inhalation with an isofluorane flow rate of 2.5 L/min and an oxygen flow rate of 1.5 L/min. During the scan, anesthesia was maintained at 0.25 L/min oxygen flow rate through a nose cone. Scans were performed at 90 kV and 0.160 mA with a 30 mm field of view for hindlimbs and a 60 mm field of view for vertebrae. For bone mineral density, total bone, lean and fat volume analysis, two consecutive scans were performed with 60 mm field of view for whole body excluding the head. The right femur was manually isolated for bone mineral density measurements. Right femur, total lean and total fat volumes were all measured using Analyze 11.0 software (Mayo Clinic) and converted to mass based on established densities. Following the scan, mice were returned to their cage and monitored for recovery in compliance with Regeneron IACUC protocols.

### Tail Suspension and Grip Endurance Test

Mice were suspended by their tails for 10 seconds and observed for any abnormal clasping phenotype. Mice were evaluated at 5, 7, and 10 weeks of age for signs of muscular deficit by their ability to hang inverted from a wire grid (wire thickness approximately 2 mm). Mice were individually placed on a wire grid that was gently shaken to prompt them to hold on as the grid was turned upside down. The time taken for the mouse to let go (up to a maximum of 60 seconds) was recorded. Mice were given three attempts to hold on as long as possible and the maximum time recorded for statistical comparison.

### Tissue Necropsy and Histology

Mice were euthanized by CO_2_ inhalation followed by cervical dislocation. The tibialis anterior (TA), quadriceps and gastrocnemius (GA) muscles were dissected and weighed. All collected muscles and organs were frozen and kept at—80°C for future examination. For histology, muscles were frozen in OCT, cryo-sectioned in cross-section at 12 μm thickness to reveal lateral and medial head, soleus, and plantaris. Adjacent sections were stained with hematoxylin and eosin, and laminin antibody. The stainings were digitally imaged using an Aperio Scanscope. Fiber size and count were determined using Spectra software. All data are expressed as the mean +/—the standard error of the mean (represented as error bars). Analysis of variance (ANOVA) was conducted using the program PRISM. Statistical significance was set at a P value less than 0.05. For skin histology, dorsal and ventral skin areas were shaved, dissected and fixed in 4% paraformaldehyde for at least 24 hours and transferred to 70% ethanol. Paraffin embedding, sectioning and hematoxylin and eosin staining on skin sections were performed by Histoserv Labs, Inc., Germantown, MD.

### Kaplan-Meier Survival Curve Analysis

Animals were observed for a period of 52 weeks and monitored for signs of morbidity according to Regeneron IACUC protocols. No mice in this study needed to be sacrificed prior to the 52-week time point based on morbidity guidelines: Mice will be euthanized if they appear to be seriously ill with any evidence of ruffled fur, lethargy, “sunken in” appearance, weight loss exceeding 20% of their starting body weights (taken typically 2 days after mice are transferred to the housing rooms from barrier), or any noticeable abnormal behavior. Survival curve and log rank test were determined using Graphpad PRISM 6 software.

## Results

### Generation of 20 lincRNA-Deleted Mouse Lines with Reporter Gene Replacement

Table 1 lists the 20 lincRNA genes on 10 different chromosomes targeted in this study and the 26 knockout deletion alleles created. We chose to mutate members of the large intergenic non-coding RNA class because, by definition, lincRNA genes are isolated from neighboring protein-coding genes and their transcripts do not overlap [6]. This feature allowed us to design deletion alleles that would have the least chance of interfering with the expression of nearby genes. We chose the targeted lincRNA genes to reflect a variety of expression patterns [7,28], with an emphasis on neural expression, and for their potential involvement in development and the regulation of gene expression [29].

Our design strategy for the lincRNA knockout mutations was guided by two goals. First, we aimed to create alleles that would accurately report the transcription activity of the lincRNA genes. Although there was evidence from cell-based and selected tissue dissection studies for tissue-specific lincRNA expression [28], we wanted to complement this knowledge base by producing the higher definition expression patterns afforded by *lacZ* expression profiling, which can resolve tissue and organ expression both spatially and temporally, thereby, revealing subdomains and in some cases, cell-type specificity not resolved by tissue dissection experiments. Second, we strove to create gene-ablating deletions that abolished the synthesis and function of the lincRNA so that any phenotypes associated with the mutations would be informative about the critical functions of the targeted RNAs.

The knockout deletions ranged in size from about 400 bp to 50 kb, with half deleting all of the annotated exons. For most of the remaining alleles, the deletion started in the second exon. The application of VelociGene methods [22] for the construction and use of large targeting vectors based on bacterial artificial chromosomes (BacVecs) was crucial to enabling us to make the large, gene ablating deletions required to ensure a null allele for this new class of large functional RNA.

Little is known about the relationship between structure and function for lincRNA genes that could guide allele design. Experience with the disruption of the Gt(ROSA)26Sor [19] and BIC (miR-155) [21] genes established that deletion and insertion after the first exon can produce reliable and tissue-specific expression of ß-galactosidase (ß-gal) or other reporters. This strategy might, however, fail to achieve a complete null mutation if the fusion transcript from the modified allele retains a functional part of the lincRNA from the 5´ portion encoded in the first exon [30]. The knockout allele designs indicated in Table 1 were therefore a compromise between the desire for a completely ablating mutation that would have the highest probability of abolishing lincRNA function and the goal of creating an allele that produced an accurate and informative gene expression profile from the ß-gal reporter. For example, for the Hotair gene we made two alleles, one that deleted nearly the entire RNA coding sequence and a second in which the deletion started in the second exon. Both alleles produced identical phenotypes (described below), but only the second functioned as a reporter of gene expression.

**Fig 1:**
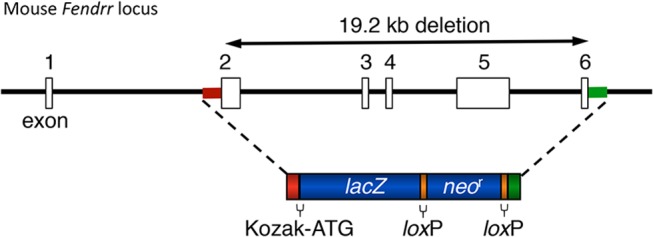
Strategy For Targeted Disruption of the *Fendrr* Gene Locus. A partial map of the mouse *Fendrr* locus is shown encompassing exons 1 to 6. Upon homologous recombination, the targeting BacVec replaced a total of 19.2 kb of the *Fendrr* genomic sequence with the *lacZ*-neomycin resistance cassette, introducing a Kozak-ATG sequence. Open boxes indicate noncoding exons. Red and green boxes on the *Fendrr* genomic locus and in the *LacZ*-neomycin resistance cassette are homologous sequences used for targeting.

For lincRNAs that reside very near a protein-coding gene and may share a divergent promoter, we set the deletion start point in the second exon to avoid the chance of disrupting the transcription of the neighboring gene. Fig 1 shows such an example for the *Fendrr* gene. The diagram shows the design elements common to all the alleles: a targeted deletion of all or most of the sequence coding for the lincRNA and replacement with a cassette that contains a sequence from the *E*. *coli lacZ* gene that encodes ß-gal and a cassette (*neo* ^r^) that expresses neomycin phosphotransferase for the selection of G418-resistant ES cell colonies. *Lox*P recombinase recognition sites that enable Cre-mediated excision prior to phenotypic analysis flank the drug selection cassette. As there is no functional open reading with which to fuse the *lacZ* sequence, each allele carries a start codon and a Kozak consensus sequence [31] for efficient translation of the ß-gal reporter.

### Specific and Diverse LincRNA Gene Expression Patterns Revealed *by LacZ* Reporter Profiling

To survey the expression patterns of the 20 targeted lincRNA genes, we applied X-gal staining for ß-gal activity on mid-gestation embryos and adult whole mount tissues and organs. The targeted lincRNA genes exhibited a variety of unique reporter gene expression patterns in both embryos and adults, representing most of the major organ systems and tissue types (Table 2). For example, in the adult tissues, expression of *Pantr2*, *Kantr*, and *Peril* was restricted to the brain; *Mannr* and *Fendrr* were expressed in lungs; *Eldr* was expressed in the urogenital system; and *Halr1* was expressed in the ribcage. One lincRNA gene, *Lincpint*, exhibited ubiquitous expression in all tissues. We did not detect expression of the *Hotair*, *Ptgs2os2*, and *Haglr* genes in any of the adult tissues we examined.

**Table 2:** Summary of *lacZ* reporter expression in embryo and adult tissue.

LincRNA	Embryo	Brain	Heart	Lungs	Liver	Ribs	Spleen	Intestine	Stomach	Kidney	Urogenital	Hindlimb
*Celrr*	+	+				+						+
*Crnde*	+	+		+		+					+	+
*Eldr*	+										+	
*Fendrr*	+			+								
*Haglr*	+											
*Halr1*	+					+						
*Hotair*	+											
*Hottip*	+							+			+	+
*HoxA11os*	+					+		+		+	+	+
*Kantr*	+	+										
*Lincenc1*	+	+										+
*Lincpint*	+	+	+	+	+	+	+	+	+	+	+	+
*Lincppara*	+	+		+		+			+		+	+
*Mannr*	+			+								
*Pantr1*	+	+				+				+		
*Pantr2*	+	+										
*Peril*	+	+										
*Ptgs2os2*	+											
*Trp53cor1*	+									+	+	
*Tug1*	+	+	+			+				+	+	

**Fig 2:**
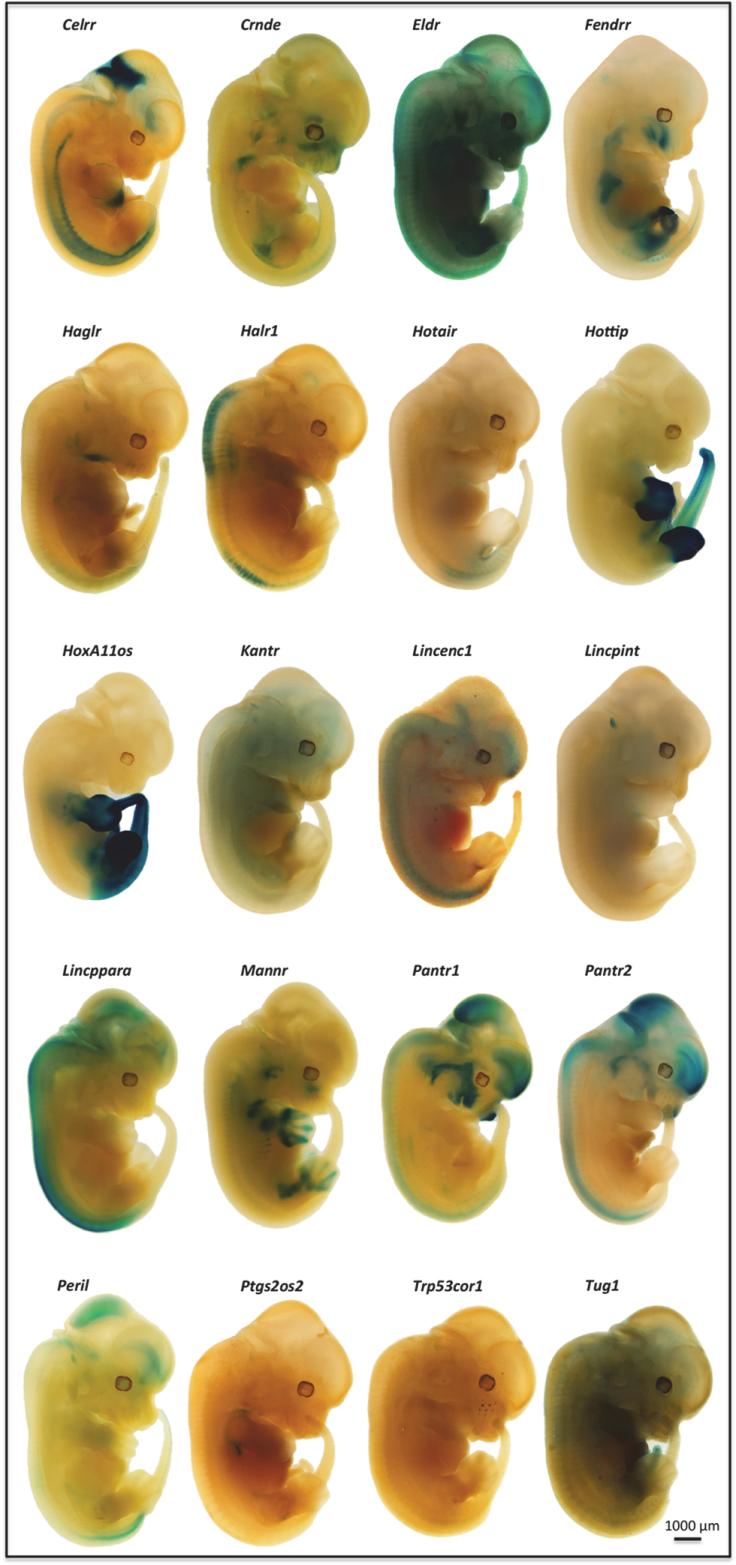
Spatial *lacZ* Reporter Gene Expression in Mid-gestation Stage LincRNA Gene-Targeted Mouse Embryos. Heterozygous E12.5 embryos fixed and stained for ß-galactosidase showed a broad range of expression (blue) of the introduced *lacZ* reporter gene in the developing brain and craniofacial region (*Pantr1*, *Pantr2*, *Celrr*, and *Peril*, see also S1 Fig), neural tube (*Pantr2*, *Halr1*, and *Lincppara*), dorsal aorta (*Celrr*), heart (*Celrr*, *Peril*, see also S1 Fig), lungs (*Fendrr*), limb buds (*Hottip*, *HoxA11os*, and *Mannr*), foregut (*Hottip*, *HoxA11os*, and *Fendrr*), posterior region and the tail (*Hotair*, *Hottip*, and *HoxA11os*). *Tug1* showed a widespread *lacZ* expression pattern, whereas expression of other reporter genes was restricted to the epidermis (*Eldr*), mammary buds (*Lincenc1*, see also S1 Fig), or the whisker placode (*Trp53cor1*, see also S1 Fig). Examples shown are representative of at least five genotype-confirmed embryos per lincRNA knockout project.

**Fig 3:**
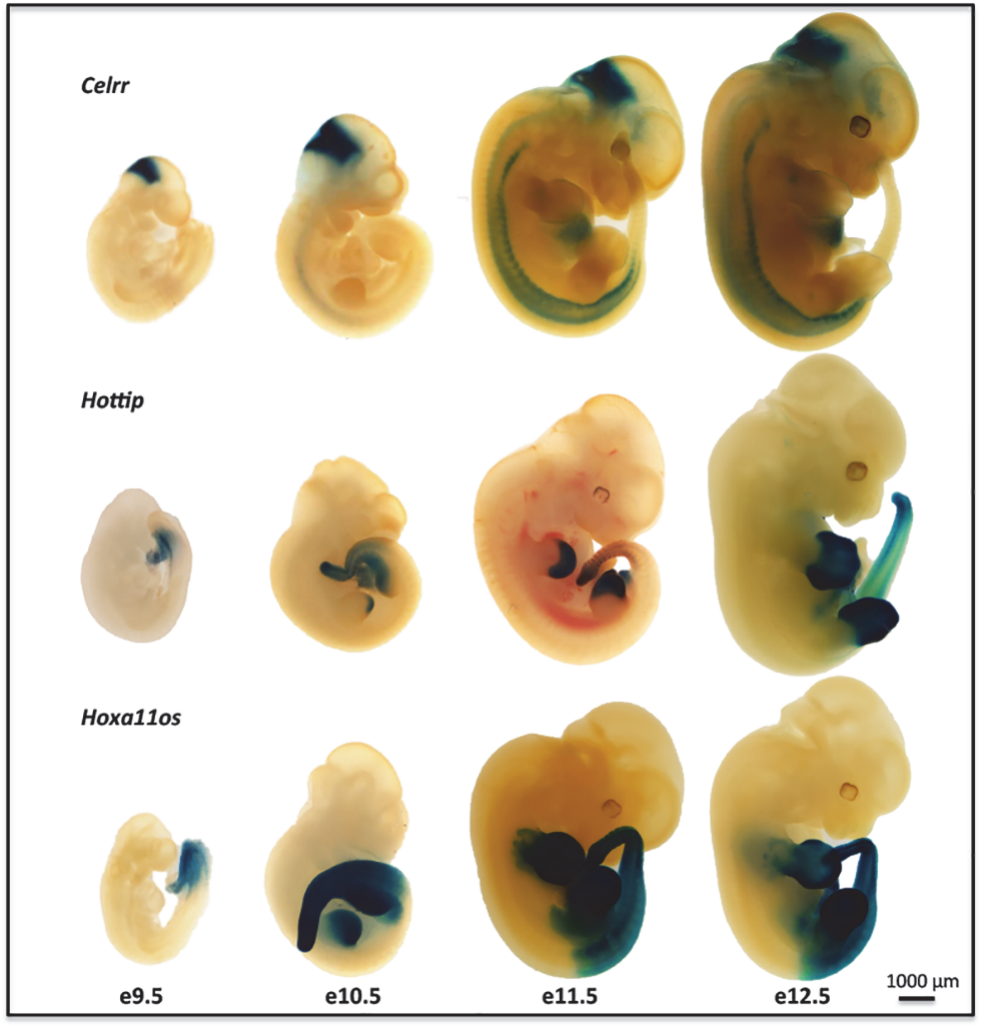
Temporal *lacZ* Reporter Gene Expression in Mid-gestation Stage LincRNA Gene-Targeted Mouse Embryos. Temporal expression patterns for *Hottip*, *HoxA11os*, and *Celrr* in F1 heterozygous embryos from the indicated stages (E9.5-E12.5) showed that expression began early at a restricted site and then extended beyond this initial site at later stages. *Celrr* expression was confined to the brain at E9.5 and progressed into the spinal cord by E12.5. *HoxA11os* expression began in the developing tail bud and progressed into the entire caudal region of the embryo, hind limb and forelimb by E12.5. *Hottip* expression also began in the developing tail bud and was then observed in the developing distal autopods of the forelimb and hind limb by E11.5 and E12.5. Examples shown are representative of at least 5, genotype-confirmed embryos per LincRNA project stained.

Embryonic expression appears to be a common feature of lincRNAs. Examination of the ß-gal reporter expression in heterozygous embryos at or around embryonic day 12.5 (E12.5) revealed a variety of specific patterns for all 20 targeted lincRNA genes (Table 2 and Fig 2). The expression profiles ranged from ubiquitous (*Tug1*) to highly specific, such as epidermal for *Eldr*, whisker placode for *Trp53cor1* (S1 Fig), or the mammary buds for *Lincenc1* (S1 Fig). The spatiotemporal patterns seen in the different extents of limb bud and tail expression for *Hottip* and *HoxA11os* are very similar to those reported for the adjacent protein-coding genes in the HoxA cluster [32,33]. The expression of *Hotair* in the posterior tail bud and genital tubercle that we observed for the ß-gal reporter was identical to that determined by *in situ* hybridization [34]. Analysis of X-gal staining at different points during embryonic development showed that for some of the lincRNA genes, expression began early at a restricted site and then extended beyond this initial locus at later stages (Fig 3), again reminiscent of Hox protein expression [35]. For example, the expression of the *Hottip* and *HoxA11os* genes began in the extreme posterior of the E9.5 embryo and then extended into the limb buds at later times. Similarly, the initial expression for *Celrr* at a site near the anterior end of the E9.5 embryos was maintained and expanded to the neural tube over the next two days.

**Fig 4:**
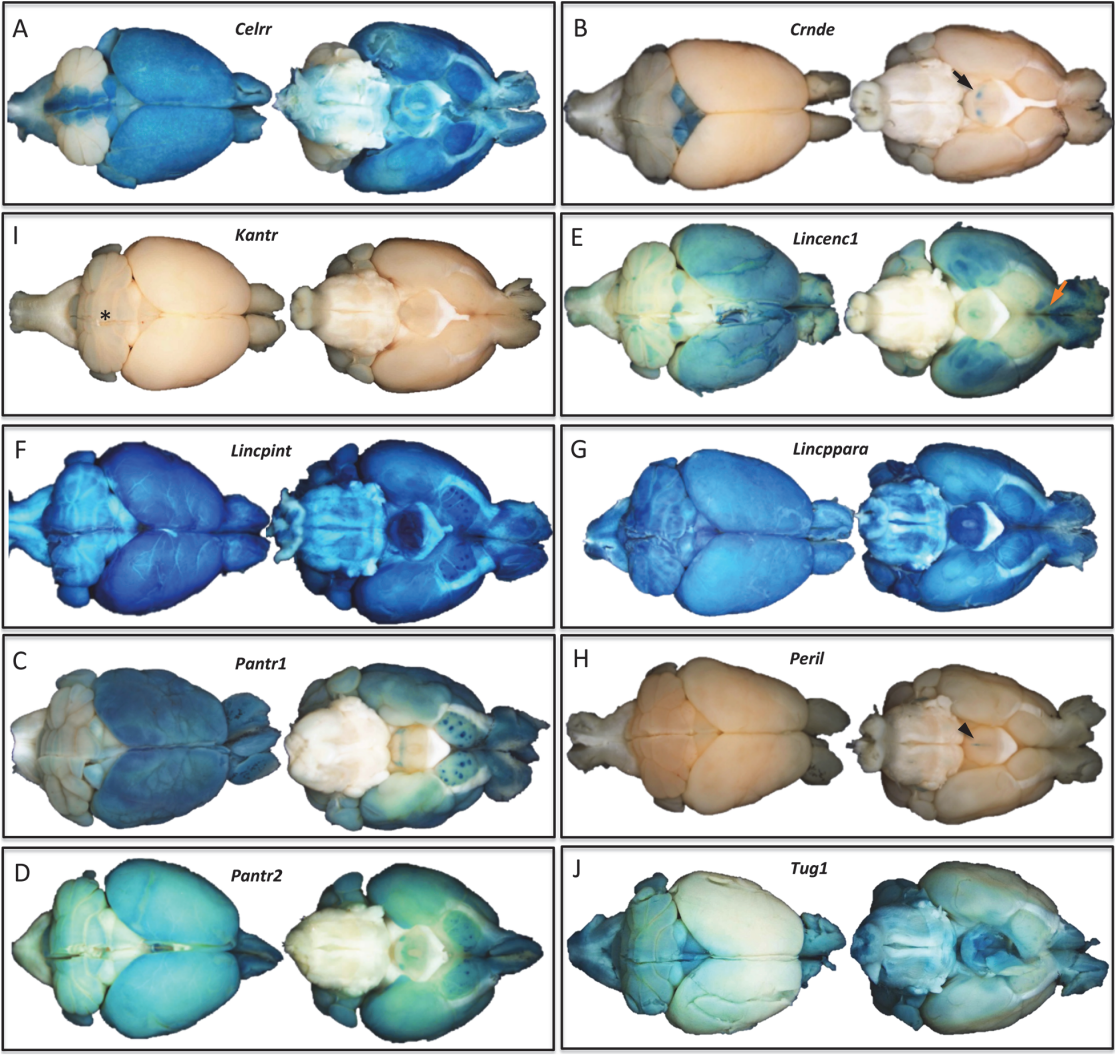
*LacZ* Reporter Expression in Brains of 6–8 Week Old lincRNA Gene-Targeted F0 Generation Heterozygotes. The brain *lacZ* expression pattern (blue) for each lincRNA gene is as follows: (A**)** *Crnde*, the colliculi (dorsal view, arrow); (B) *Pantr1*, neocortex, olfactory bulb, basal forebrain, and hypothalamus; (C**)** *Pantr2*, neocortex, olfactory bulb, cerebellum, hypothalamus, and basal forebrain; (D**)** *Lincenc1*, neocortex, parts of the cerebellum and medial hypothalamus, especially strong patterning in the olfactory projection and olfactory projection areas of the temporal cortex (ventral view, red arrow); (E**)** *Celrr*, broadly in gray matter with the exception of the lateral cerebellum and ventral pons; (F) *Kantr*, possibly in deep cerebellar layers (dorsal view, star); (G**)** *Lincpint*, ubiquitously in gray matter, especially intense in the hypothalamus; (H**)** *Lincppara*, ubiquitously in gray matter, especially dense in the hypothalamus; (I**)** *Peril*, midline of the hypothalamus (ventral view, arrowhead); and (J) *Tug1*, spinal cord gray matter and light gray matter in most structures except for the neocortex. n = 2, genotype confirmed male mice per lincRNA knockout project.

Consistent with the frequent brain expression seen among human tissue-specific lncRNAs [5], we found that half of the 20 targeted mouse lincRNA genes were transcriptionally active in the adult brain. As with the embryonic lincRNA expression, the brain patterns (Fig 4) were unique and varied from ubiquitous (*Lincppara* and *Lincpint*) to highly restricted specific brain structures (*Peril*, *Crnde*, and *Kantr*).

### *Lincpint’s* Unique Increased Expression with Age Correlates with Aging-Related Phenotypes

**Fig 5:**
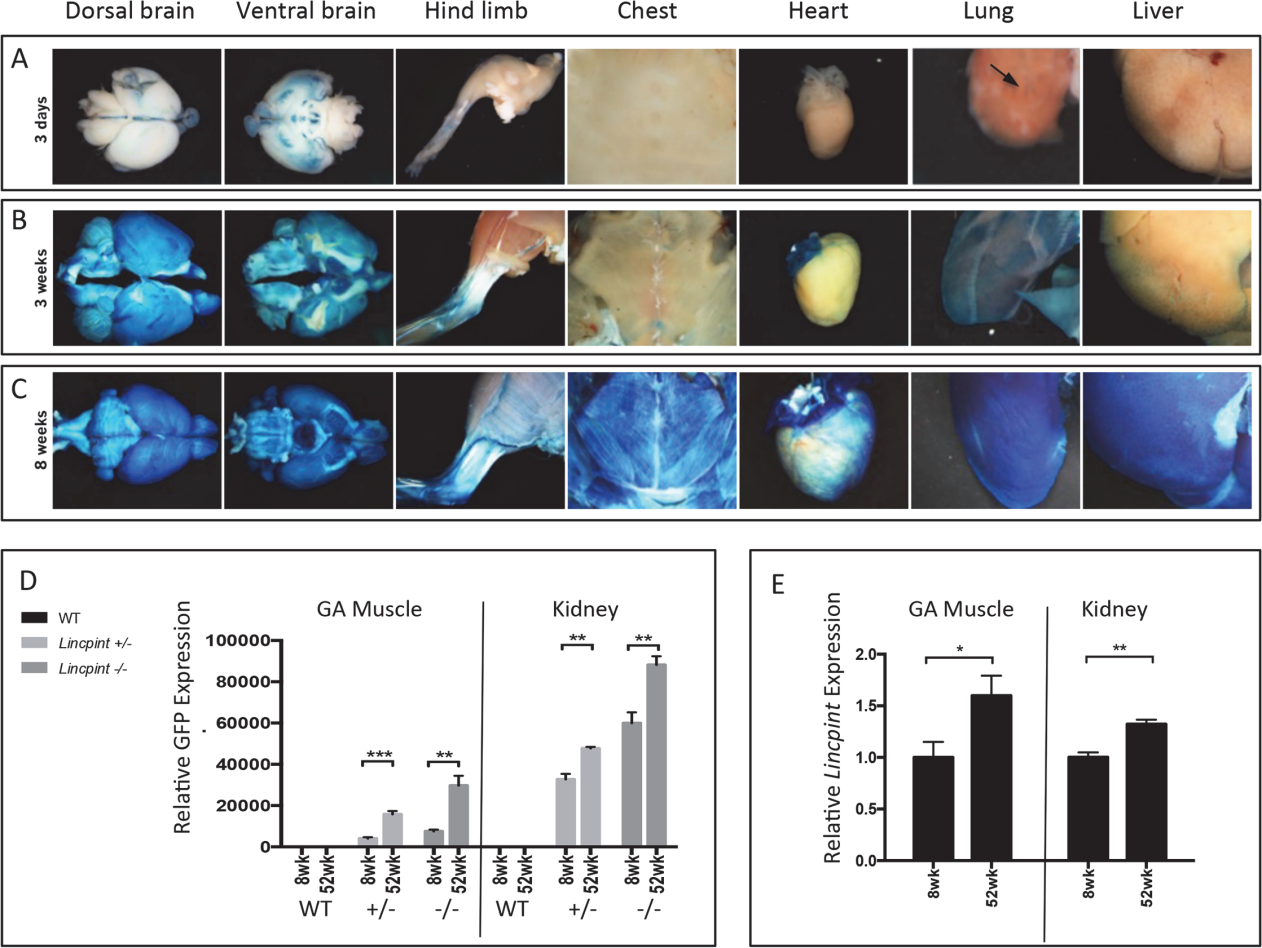
Increased Expression of *Lincpint* from Postnatal Day 3 to 8 Weeks of Age. *LacZ* reporter expression (blue) at 3 days, 3 weeks, and 8 weeks in F0 heterozygotes shows increasing *Lincpint* expression with age. (A) At 3 days, ßgalactosidase staining is only observed in portions of the brain, tendons and ligaments of the hind limb, and some bronchioles in the lung (arrow). (B) At 3 weeks, there is increased staining in the brain, hindlimb, atria of the heart, lung, and liver. (C) By 8 weeks of age, the whole brain, skeletal muscle of the hindlimb and chest, atria and myocardium, lung, and liver tissue all exhibit strong ß-galactosidase staining representative of increased *Lincpint* expression. Examples shown are representative of n>4 mice per group. (D) RT-PCR analysis of *GFP* in GA (gastrocnemius) muscle and kidney isolated from 8 week-old and 52 week-old wild type (WT), heterozygous (*Lincpint-GFP* ^*+⁄−*^) and homozygous (*Lincpint-GFP* ^*−⁄−*^) mice. (E) Comparison of endogenous *Lincpint* RNA level in GA (gastrocnemius) muscle and kidney of 8 week-old versus 52 week-old wild type (WT) tissues demonstrating increased in *Lincpint* gene expression. Examples shown are representative of n>4 mice per group.

Of the 20 lincRNA genes targeted, only *Lincpint* showed a global whole-body expression pattern, mostly restricted to postnatal life (Table 2). Unique to *Lincpint*, we observed an increase in its expression with age (Fig 5). In 3-day old neonates, *Lincpint* transcription activity was low (brain) or undetectable (ribcage muscle) but then gradually appeared in 3-week old mice and became strong and ubiquitous by 8 weeks of age. Although the strength and timing of *Lincpint* expression varied among different organs and tissues, the general trend was a steady increase in expression after birth to a plateau in adulthood. Because cytochemically detectable ß-gal at pH 6.0 has been reported to increase during the replicative senescence of fibroblast cultures and has been used as a marker of cellular senescence [36], we generated *Lincpint-GFP* (green fluorescent protein) mice as a second reporter line to further characterize age-associated change in *Lincpint* expression independent of ß-gal. We observed a similar age-dependent increase in GFP expression during embryonic development and in adult tissues of heterozygous (*Lincpint-GFP* ^+⁄−^) and homozygous (*Lincpint-GFP* ^−⁄−^) mice (Fig 5D and S1 Fig and data not shown). By RT-qPCR analysis, GFP mRNA levels in skeletal muscle and kidney of *Lincpint-GFP* ^+⁄−^ mice was half that of *Lincpint-GFP* ^−⁄−^ mice. Analysis of endogenous mouse *Lincpint* RNA in multiple tissues harvested from young (8 week-old) and aged (52 week-old) wild-type (WT) mice also showed a similar age-associated increase in gene expression (Fig 5E and data not shown). To our knowledge, this age-related dynamic expression pattern is novel. We have not observed a similar profile in our experience of *lacZ* profiling experiments for hundreds of protein-coding genes [22].

**Fig 6:**
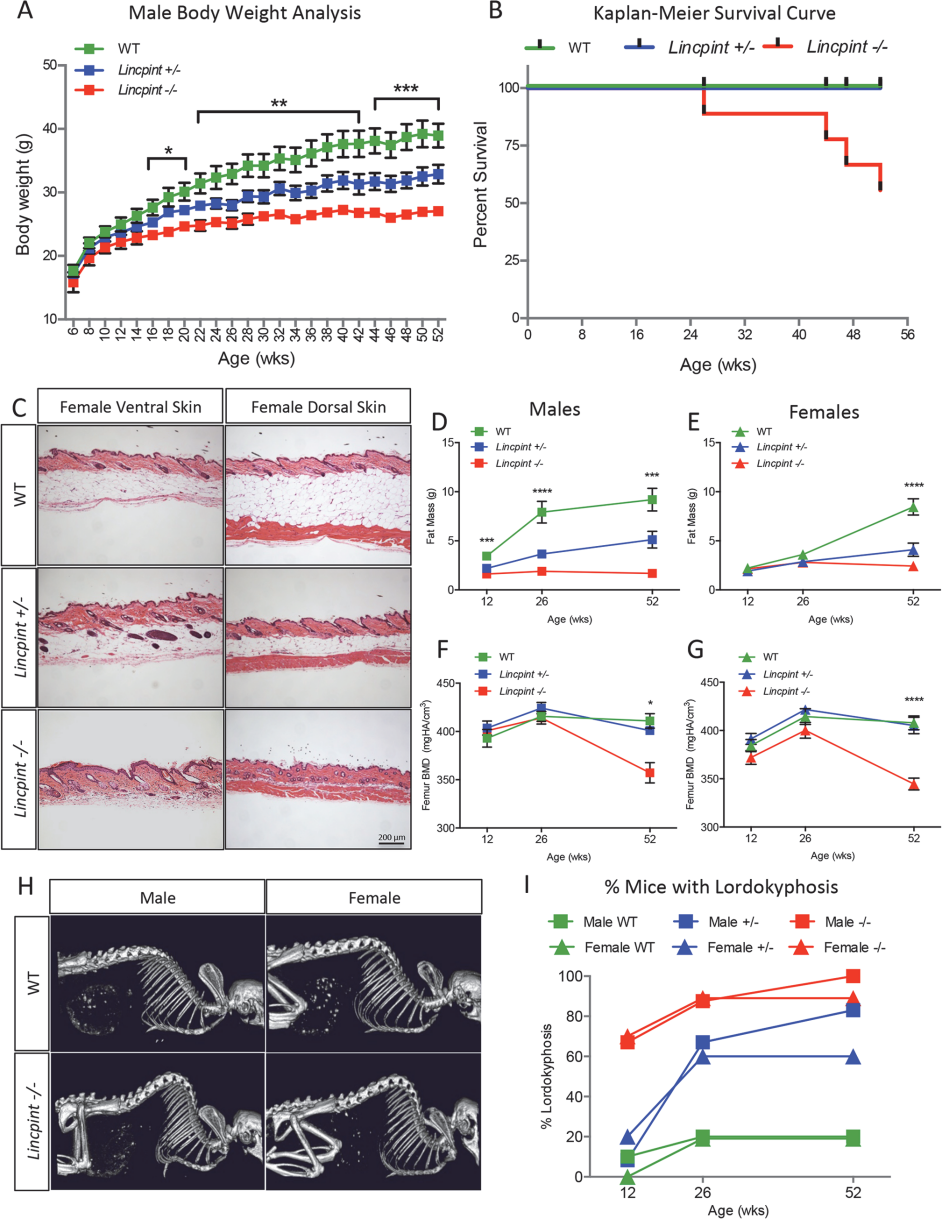
Aging-associated Phenotypes in *Lincpint* Knockout Mice. (A) *Lincpint* ^*−⁄−*^ and *Lincpint* ^*+⁄−*^ male mice exhibit a significantly slower growth rate than their wild type (WT) littermates and begin to show significant weight loss near 6 months of age. Data are plotted as the mean +/− SEM, n > 9 mice for each group. Significance was assessed by a one-way ANOVA (*, *P* < 0.05; **, *P* < 0.005; ***, *P* < 0.001). (B) Kaplan-Meier analysis of homozygous with heterozygous and WT mice. *Lincpint* ^*−⁄−*^ male mice exhibit a significant reduction in survival compare to *Lincpint* ^*+⁄−*^ and wild type littermates. Data are plotted as percent survival over 1 year observation. (C) Ventral and dorsal skin sections in *Lincpint* ^*−⁄−*^ mice compared with *Lincpint* ^*+⁄−*^ and WT littermates. (D, E, F, and G) MicroCT evaluation of body composition at 12-, 26- and 52-weeks of age. (D, E) Male *Lincpint* ^*−⁄−*^ and *Lincpint* ^*+⁄−*^ mice exhibit a significant reduction in body fat as early as 26-week of age. Female *Lincpint* ^*−⁄−*^ mice have reduced body fat at an older age noticeably at 52-week of age (***, *P* < 0.001, one-way ANOVA). (F, G) A significant reduction in femur bone mineral density (BMD) observed in both males and females *Lincpint* ^*−⁄−*^ compared with their *Lincpint* ^*+⁄−*^ and WT littermates (*, *P* < 0.05; ***, *P* < 0.001, one-way ANOVA). (H) MicroCT images depict pronounced lordokyphosis (curvature of the spinal column) seen in older male and female *Lincpint* ^*−⁄−*^ mice compared with WT littermates. (I) Approximately 70% (6/9 males and 7/10 females) of *Lincpint* ^*−⁄−*^ mice have lordokyphosis by 12 weeks of age, compared with 0–20% of *Lincpint* ^*+⁄−*^ (1/12 males and 2/10 females) and WT (1/10 males and 0/11 females) littermates. By 26 weeks of age the proportion of *Lincpint* ^*−⁄−*^ mice with lordokyphosis increased to nearly 90% (7/8 males and 8/9 females) and appeared in approximately 60% (8/12 males and 6/10 females) of *Lincpint* ^*+⁄−*^ mice, compared with less than 20% (2/10 males and 2/11 females) of WT littermates. n ≥ 9 mice per group for all observations reported.

The striking age-related increase in whole-body *Lincpint* expression revealed by *lacZ*, *GFP* and RNA profiling (Fig 5) suggested a global homeostatic role for *Lincpint* in the maintenance of normal health as mice age. To test this hypothesis we bred the *Lincpint* knockout mouse line to homozygosity and conducted a longitudinal study comparing homozygous (*Lincpint* ^−⁄−^) mice with WT and heterozygous (*Lincpint* ^+⁄−^) littermate controls. The *Lincpint* ^−⁄−^ mice appeared healthy and normal at birth; however, at the age of 3 months they began to show signs of an early onset aging-like phenotype. Body weight measurements revealed that both male and female *Lincpint* ^−⁄−^ mice exhibited a slower growth rate compared with their WT littermates, but it was more pronounced in the males (Fig 6A). By one year of age, male *Lincpint* ^−⁄−^ mice were more than 30% lighter and *Lincpint* ^+⁄−^ mice were 15% lighter than their WT littermates, whereas *Lincpint* ^−⁄−^ females were 27% lighter (data not shown). Kaplan-Meier analysis comparing homozygous with heterozygous and WT male mice (Fig 6B) demonstrated that the loss of *Lincpint* is associated with poor survival outcome. We found no sign of tumors or lesions in the mutant mice as they aged, but some *Lincpint* ^−⁄−^ mice developed herniation, including protrusion of the xiphoid process on the chest associated with thinning of the abdominal wall (data not shown). There was an age-dependent abnormal hindlimb clasping posture when the mice were suspended from their tails (data not shown). The severity of this phenotype varied, but its frequency increased progressively with age, suggesting a decline of muscle strength. We also observed fur loss in both male and female mice (data not shown). Histological analysis of skin sections collected from the ventral and dorsal bodies of *Lincpint* ^−⁄−^ mice revealed fibrosis and a noticeable difference in hair follicle development along with a dramatic reduction in the thickness of the subcutaneous fat layer (Fig 6C).

Non-invasive whole body analysis by X-ray microtomography (microCT) of individual mice as they aged indicated a significantly lower fat content in male (Fig 6D) and female (Fig 6E) *Lincpint* ^−⁄−^ mice compared with their WT littermates. The loss of total body fat was likely the major contributor to the decline in body weight as they age (Fig 6A). The *Lincpint* ^−⁄−^ mice also had a significantly lower femur bone mineral density than WT (Fig 6F and 6G). Male mice had significantly decreased lean mass at 52 weeks of age. Both males and females showed significantly decreased muscle mass for the gastrocnemius complex (GA) and tibialis anterior (TA) beginning at 26 weeks of age (not shown). Skeletal imaging revealed the appearance of severe lordokyphosis in both male and female *Lincpint* ^−⁄−^ mice compared with WTs (Fig 6H). Approximately 70% (6/9 males and 7/10 females) of 12-week old *Lincpint* ^−⁄−^ mice displayed lordokyphosis and approximately 95% (9/9 including those that died before the 52 week time-point, or 6/6 surviving males and 8/9 females) by 52 weeks of age (Fig 6I). In contrast, less than 20% (2/10 males and 2/11 females) of 26-week old WT mice displayed slight lordokyphosis and this frequency did not increase with age. *Lincpint* ^+⁄−^ mice did not develop significant lordokyphosis until 26 weeks of age, indicating an unusual age-dependent haploinsufficiency for *Lincpint*. The spectrum of age-associated pathologies in the *Lincpint* knockout mice suggests that *Lincpint* may be important for the maintenance of health and the avoidance of pre-mature aging during the normal life span of the mouse.

### Loss of *Fendrr* Causes Perinatal Lethality as the Result of Respiratory Distress

**Fig 7:**
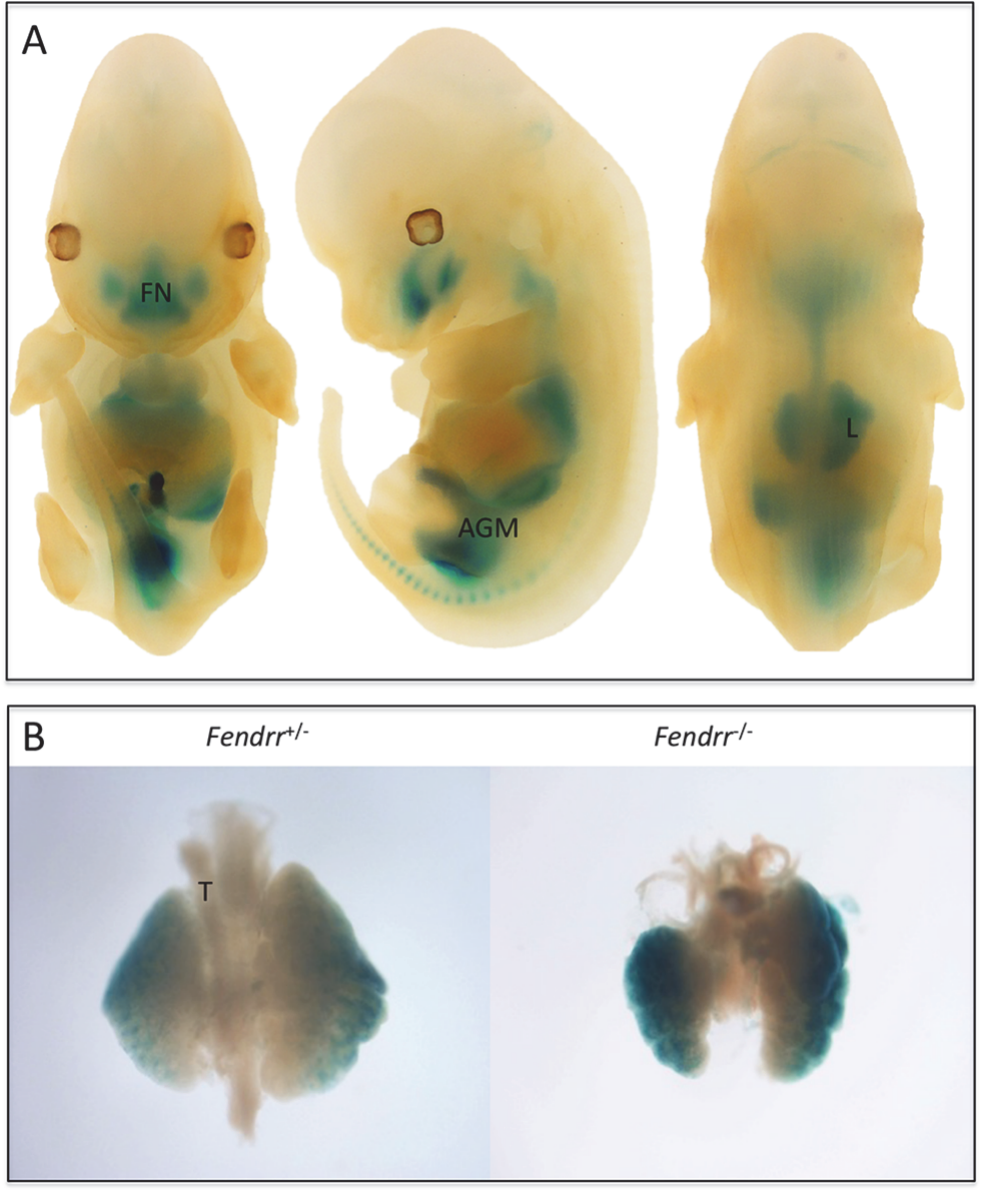
Abnormal Lung Morphology in *Fendrr* Knockout Mice at E13.5. (A) *LacZ* reporter gene expression at E12.5 in *Fendrr* ^*−⁄−*^ embryos in the frontonasal region (FN) of the face, the aorta gonad mesonephros (AGM) region, and the respiratory tract, including the lungs (L) and trachea (T). (B) Dissection of lungs at E13.5 revealed an abnormal, disorganized, globular phenotype in the lobes of *Fendrr* ^*−⁄−*^ embryos compared with *Fendrr* ^*+⁄−*^.

Of the 20 lincRNA knockout mouse lines, *Peril* ^−⁄−^ and *Fendrr* ^−⁄−^ mice showed perinatal lethality [29]. Our *Fendrr* knockout allele has a 26 kb deletion from exon 2 to the last annotated exon (Fig 1). X-gal staining of E12.5 homozygous embryos showed *lacZ* expression in the frontonasal process, upper respiratory tract, lungs, and the posterior Aorta-Gonad-Mesonephron (AGM) region (Fig 7A) that was identical in both heterozygous (not shown) and homozygous embryos, indicating grossly normal organogenesis. An isolated look at the developing lungs at E13.5 revealed defects in the knockout embryos: the lungs were small and the lobes appeared globular and disorganized (Fig 7B). Mice homozygous for deletion of the *Fendrr* gene survived to birth but succumbed shortly after from apparent breathing problems. The *Fendrr* mutant perinatal lethal phenotype was identical in mice on 2 different genetic backgrounds: a C57BL6/129 hybrid reported here and in mice further backcrossed to C57BL/6 in a separate breeding program [29].

### Loss of *Hotair* and *Hottip* Causes Morphological and Functional Defects in Skeleton and Muscle

**Fig 8:**
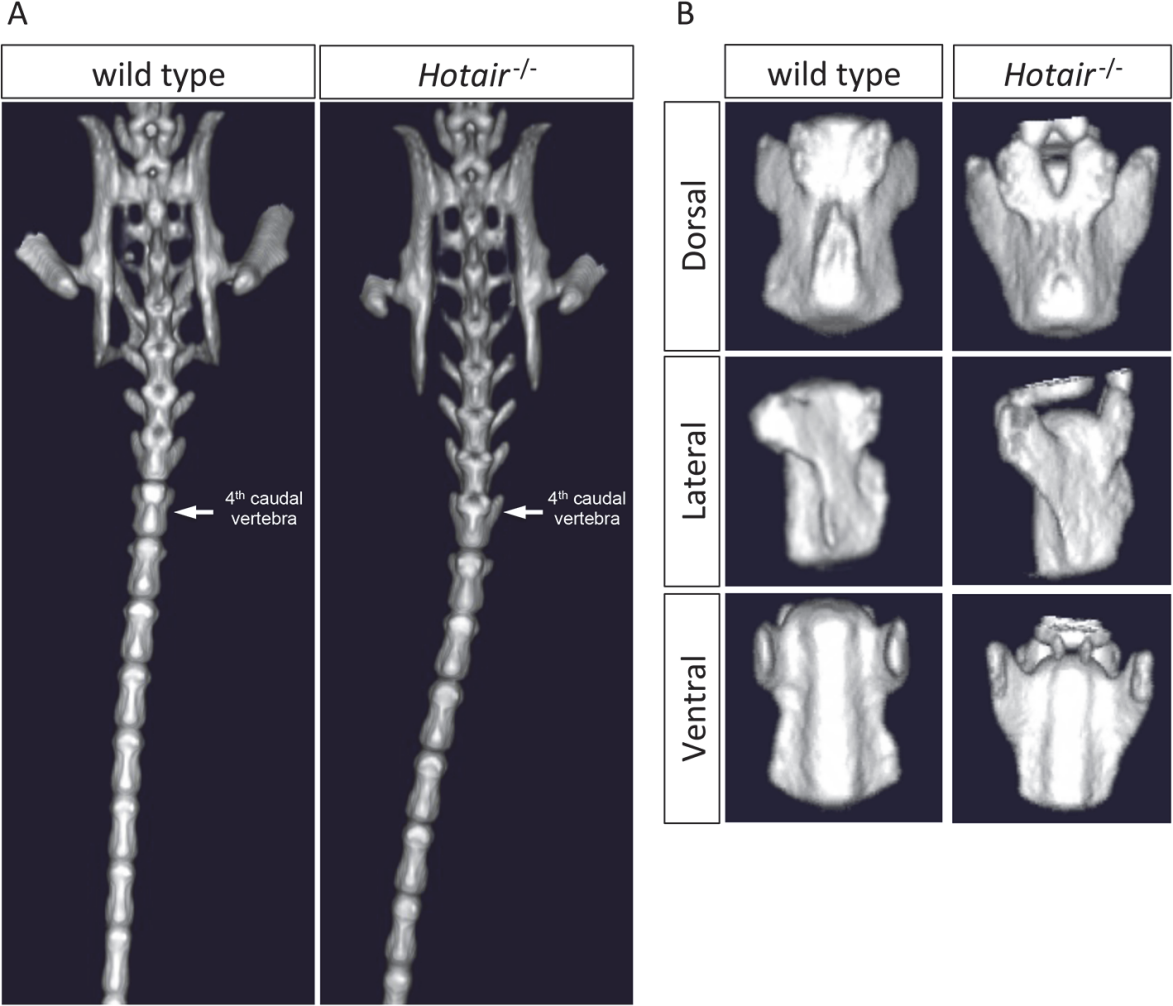
Homeotic Transformation Observed in the 4th Caudal Vertebra of *Hotair* ^*−⁄−*^ mice. (A) Visualization of the sacral and caudal region of the mouse skeleton by microCT reveals a homeotic transformation in *Hotair* ^*−⁄−*^ mice of the 4th caudal vertebra to a structure similar to that of the 3rd caudal vertebra. (B) Dorsal, lateral and ventral comparison of WT and *Hotair* ^*−⁄−*^ 4th caudal vertebra reveals a structural abnormality in homozygotes indicative of a homeotic transformation.

**Fig 9:**
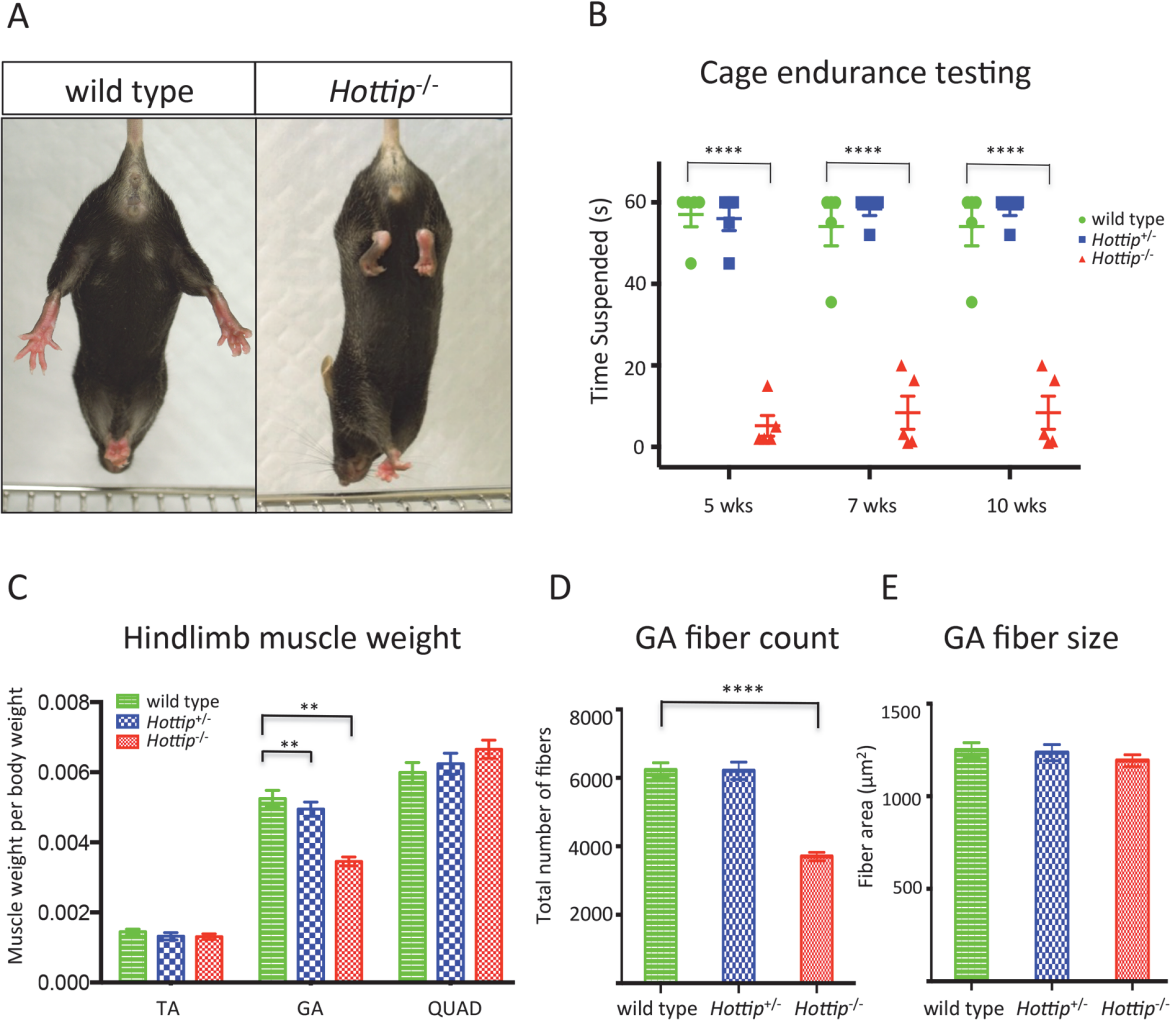
Abnormal Hindlimb Posture, Reduced Grip Strength, and Muscle Wasting in *Hottip* ^*−⁄−*^ Mice. (A) *Hottip* ^*−⁄−*^ mice demonstrated unusual hindlimb clasping posture when suspended by the tail. (B) Cage endurance testing revealed that *Hottip* ^*−⁄−*^ mice have a reduced ability to remain suspended from an inverted wire cage top, n = 5 mice for each group. (C) The right and left TA (tibialis anterior), GA (gastrocnemius) and Quad (quadriceps) muscles from WT, *Hottip* ^*+⁄−*^ and *Hottip* ^*−⁄−*^ mice were weighed. Muscle weights are normalized to body weight and calculated to include both right/left muscle weights. Data are means +/−SEM, n = 6 mice for each group. A significant decrease in muscle weight was observed only in the GA of *Hottip* ^*−⁄−*^ animal in both males and females (male data not shown). Asterisks indicate a significant difference in the *Hottip* ^*−⁄−*^ GA muscle weights compared to all other control groups (P< 0.01). (D) Comparison of GA muscle fiber numbers in WT, *Hottip* ^*+⁄−*^ and *Hottip* ^*−⁄−*^. A significant reduction of fiber count was observed in *Hottip* ^*−⁄−*^. Significance assessed by one-way ANOVA (P < .0001 (E) Comparison of mean cross-sectional area of muscle fibers. Cross sections taken from the GA muscle were stained with an antibody against laminin and measured. There is no noticeable size difference between *Hottip* ^*−⁄−*^ and control skeletal muscles, n = 6 mice per group for all muscle analyses.

Embryonic X-gal staining for the *Hotair* and *Hottip* genes showed restricted expression in the posterior and distal limb buds (Fig 2). Consistent with these developmentally restricted expression patterns, deletions of the *Hotair* and *Hottip* genes caused morphological malformations in the tail and hind limbs of adult mice. In *Hotair* ^−⁄−^ mice we observed an apparent homeotic transformation of the 4th caudal vertebra, which became anatomically similar to the 3rd caudal vertebra (Fig 8). The *Hottip* ^−⁄−^ mice displayed an abnormal hindlimb clasping posture when suspended from their tails compared with wild type littermates (Fig 9A). This behavioral abnormality was accompanied by a loss in grip endurance as measured by a test in which the mice are challenged to remain suspended on an inverted wire cage. Wild type and *Hottip* ^+⁄−^ mutants held on for approximately one minute, while their homozygous littermates released their grip within 10–20 seconds (Fig 9B). This apparent reduction in grip strength was associated with a loss of muscle mass for the gastrocnemius but not for the tibialis anterior or the quadriceps muscles (Fig 9C). We observed an approximate 40% reduction in the number of muscle fibers in the gastrocnemius but no reduction in average fiber size (Fig 9D and 9E). In addition to the muscle defects in the *Hottip* knockout mice, we also found a hindlimb skeletal malformation: a shortening in the length of the calcaneum bone (S2 Fig).

## Discussion

In the past several years there has been an explosion in our understanding of the non-protein-coding component of the genome, especially in mammals. In addition to long-recognized classes of non-coding functional RNAs such as ribosomal, transfer, small nuclear, small nucleolar, small cytoplasmic RNAs, the RNA components of the RNase P, RNase MRP, and telomerase enzymes and the more recently discovered microRNAs and the PIWI-associated piRNAs, we can now include at least 15,000 members of the long non-coding RNA class [2–6]. As we begin to understand the genomic presence and expression of lncRNA genes, the next goal is to discover their biological functions. As a first step to tackling this challenge, we applied mouse gene targeting technology, the most powerful tool for the determination of mammalian gene function, to create a resource of knockout mouse lines for 20 lincRNA genes [29].

Structure-function relationships for the lincRNAs are poorly understood. For this reason, it was crucial in this initial study to create knockout alleles with deletions that removed most if not all of the lincRNA coding potential to have the highest probability of creating a loss of function mutation. The ambiguous and incomplete annotation of many lincRNA loci, with multiple reported transcripts perhaps generated by alternative splicing or transcription initiation sites, adds to the difficulty of knockout allele design. New understanding of the molecular characteristics important for lincRNA function should inform the design of the next generation of lincRNA alleles with more precisely directed modifications of sequences critical to function and also permit advanced and flexible conditional strategies.

A key goal of our lincRNA knockout survey was to create alleles that in addition to abolishing function also reported the gene’s spatiotemporal pattern of expression. Despite not having a protein coding open reading frame as a guide, we were successful in designing alleles that reported gene expression for all of the 20 targeted genes. One of the alleles that produced no *lacZ* expression in the adult stage was *Ptgs2os2* (see Fig 2 and S1 Fig for embryonic expression), which is known to be one of the lincRNAs most strongly induced by inflammatory signals [6,37]. The *Ptgs2os2* knockout line should prove a valuable resource for studies of how a lincRNA’s expression responds to infection or other inflammatory insults and what biological role it plays in the process.

One of the criteria we applied in our selection of which lincRNA genes to target for this survey was an expectation of expression in neural tissues. Ten of the targeted genes showed *lacZ* reporter expression in the adult brain and each exhibited a unique pattern (Fig 4), ranging from strong whole brain expression (*Lincpint*) to light grey matter expression in most structures *(Tug1*) to highly restricted expression exclusive to the colliculi (*Crnde*) or the midline of the hypothalamus (*Peril*). The variety and specificity of the gene expression patterns in the brain was also evident in other tissues and was similar to those we have seen with reporter alleles for protein-coding genes. Our lincRNA gene *lacZ* expression profiling patterns were consistent with the tissue-specific expression found by RNA quantification experiments in wild type mouse tissues [29]. Prior to this study, however, the exquisite tissue and cell type specificity of lincRNA gene expression was not appreciated because previous quantification methods could not deliver the high definition and cell-type resolution of *lacZ* reporter profiling (Fig 2).

Embryonic expression was a feature shared by all the lincRNA genes we examined. *LacZ* profiling delivered a high definition view of whole embryos that revealed the broad range of specific patterns unique to each lncRNA. Examples include the exquisitely specific expression observed in the whisker placode for *Trp53cor1* and the mammary bud for *Lincenc1*, the epidermal expression of *Eldr*, the limb bud expression of *Hottip* and *HoxA11os*, and the ubiquitous expression of *Tug1* (Figs 2 and 3 and S1 Fig). These varied patterns might point to a common role for lincRNAs in the regulation of key events in development.

Another value of *lacZ* profiling is that it can guide and focus the design of phenotypic studies. For example, the highly restricted posterior expression patterns for heterozygote *Hotair* and *Hottip* embryos suggested that we might find knockout phenotypes in posterior body parts. Consistent with this expectation, we observed an apparent homeotic transformation of the 4th caudal vertebra in *Hotair* ^−⁄−^ mice (Fig 7), and we found abnormalities of the hind limbs that included muscle weakness and skeletal malformations in *Hottip* ^−⁄−^ mice (Fig 9 and S2 Fig). The *Hotair* homeotic phenotype has also been observed in mice with a different *Hotair* knockout allele [38]. We found that expression of *Fendrr* in heterozygotes was restricted to the lungs in adult mice (Table 2) and prominent in the developing respiratory tract in embryos (Fig 2). Perhaps not surprisingly, *Fendrr* homozygotes exhibited respiratory stress and subsequent perinatal death due to defective structural maturation of the lungs. Our *Fendrr* knockout phenotype resembles the rare human lethal lung development disorder alveolar capillary dysplasia with misalignment of pulmonary veins (ACD/MPV), in which patients exhibit a deficiency in lung lobe development and suffer postnatal respiratory distress within minutes to hours after birth [39]. At least one ACD/MPV patient was reported to have an 11 kb deletion within the *FOXF1-AS1* gene, the human homolog of mouse *Fendrr*, expressed in normal newborn human lungs [40]. Grote *et al*. [18] reported a mutant mouse with a modification of the *Fendrr* gene that produced lethality at around E13.75 associated with a prominent omphalocele, reduction in ventral body wall thickness, and a heart defect causing blood accumulation in the right atrium. We did not observe any of these phenotypes. The discrepancies between the phenotypes may be explained by the different allele designs. Our allele deleted *Fendrr* exon 2 to the end, designed to avoid disruption of the promoter that could be shared with the adjacent *Foxf1* protein-coding gene. The *Fendrr* allele of Grote *et al. [18]* consisted of the insertion of a transcriptional stop element in the first exon and did not include a reporter gene.

The most remarkable adult expression pattern we observed was for *Lincpint*, which exhibited an increase in the extent and intensity of X-gal staining and GFP as the mice aged from newborn to mature adults (Fig 5). Consistent with this finding, we detected an upregulation of the endogeneous lincpint RNA expression in aged mice (Fig 5D). This striking age-associated expression pattern prompted us to conduct a longitudinal analysis for growth rate and overt signs of abnormal health. Compared with WT mice, we found that as the *Lincpint* ^*−⁄−*^ mice aged, they exhibited progressive hair loss and signs of muscle weakening, severe lordokyphosis, reduced body fat and bone mineral density, a slower growth rate, and reduced survival. Surprisingly, these results were replicated in the heterozygous mice, but to a lesser extent. This spectrum of age-associated phenotypes, along with the unusual increase in gene expression with age, implies that mice may require a critical dose of *Lincpint* for the general maintenance of health and tissue function during the normal life span, and for the first time points to potential role of LincRNA in physiological aging. A recent study showed that *Lincpint* is a direct target for p53, providing a link between the p53 pathway and epigenetic silencing by the polycomb repressive complex 2 (PRC2) [41]. A growing body of evidence has implicated the critical role of p53 in cellular senescence and the control of aging. It will be of great interest to investigate the regulation of *Lincpint* and its potential involvement in p53-dependent cellular senescence and aging [42]. This could reveal key mechanisms in the physiological aging process in mammals with potential clinical implications in human diseases including those associated with aging and cancer.

Our aim in initiating this work was not only to shed light on the functions of the 20 particular lincRNAs whose genes we chose to mutate, but also to obtain a better understanding of the general properties of lncRNAs as a class. This collection could serve as a seed for a larger-scale effort to mutate many more members of the lincRNA gene family. Many lincRNAs have been shown to be associated with proteins that participate in the regulation of transcription at the chromatin level. This might suggest a broad, general, and redundant function in gene expression much like the interplay of miRNAs in the maintenance of tissue-specific gene expression profiles at the post-transcriptional level. Our results, however, appear to point in a different direction. The unique phenotypes and exquisitely specific expression patterns described here and in Sauvageau et al. [29] argue for specific, direct, and determinative functions for lincRNAs. Although this study is only the beginning of the analysis of this collection of knockout mice, it reveals lincRNAs as a new class of functional encoded molecules that, like proteins, serve diverse roles in the embryonic development, physiology, and homeostasis of a broad array of tissues and organs in mammals.

## Supporting Information

S1 ARRIVE Guidelines Checklist Completed “The ARRIVE Guidelines Checklist” for reporting animal research experiments in this manuscript. (DOCX)

S1 Fig Specific Mid-Gestational *lacZ* Expression Profiles for *Peril*, *Ptgs2os2*, *Trp53cor1* and *Lincenc1*. (A) *LacZ* reporter profiling for *Peril* shows a specific neuronal expression pattern as well as strong expression in the heart and posterior tail region. (B) *Ptgs2os2 lacZ* reporter expression is restricted to the base of developing forelimbs and hindlimbs. (C) *Trp53cor1 lacZ* reporter expression is specific to the developing whisker placode in the nasal process. E12.5 embryos collected from the same litter capture the progression of whisker placode development over a short period of time. (D) Forelimbs and hindlimbs were removed in the *Lincenc1* ^+⁄−^ embryos to reveal mammary bud expression (arrowheads). Ventral view of E12.5 *Lincenc1* ^+⁄−^ embryo: *lacZ* expression is detected in five pairs of mammary buds.
(TIF)

S2 Fig Skeletal Malformations Observed in *Hottip* Mutant Mice. In addition to a skeletal muscle phenotype in the hindlimb, *Hottip* ^−⁄−^ mice also display a skeletal bone abnormality visualized by 3D microCT. Both male and female (C and F) *Hottip* ^−⁄−^ mice have shortened calcanea (arrows) in comparison to (A and D) WT and (B and E) *Hottip* ^+/−^ littermate controls.
(TIF)
