# Supplementary material for: Diverse Phenotypes and Specific Transcription Patterns in Twenty Mouse Lines with Ablated LincRNAs
Source: PLoS One. 2015 Apr 24;10(4):e0125522. doi: 10.1371/journal.pone.0125522 (PMC4409293; doi:10.1371/journal.pone.0125522)

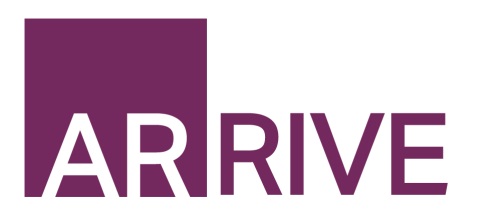


The ARRIVE Guidelines Checklist

Animal Research: Reporting In Vivo Experiments

Carol Kilkenny^1^, William J Browne^2^, Innes C Cuthill^3^, Michael Emerson^4^ and Douglas G Altman^5^

*^1^The National Centre for the Replacement, Refinement and Reduction of Animals in Research, London, UK, ^2^School of Veterinary Science, University of Bristol, Bristol, UK, ^3^School of Biological Sciences, University of Bristol, Bristol, UK, ^4^National Heart and Lung Institute, Imperial College London, UK, ^5^Centre for Statistics in Medicine, University of Oxford, Oxford, UK.*

|  | | ITEM | RECOMMENDATION | Section/ Paragraph |
| --- | --- | --- | --- | --- |
| 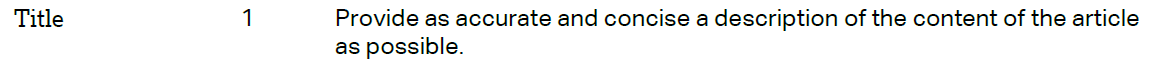 | | | Title |  |
| 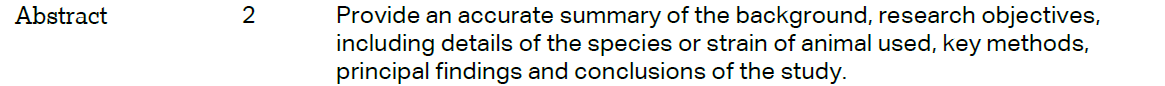 | | | Abstract |  |
| INTRODUCTION | | |  |  |
| 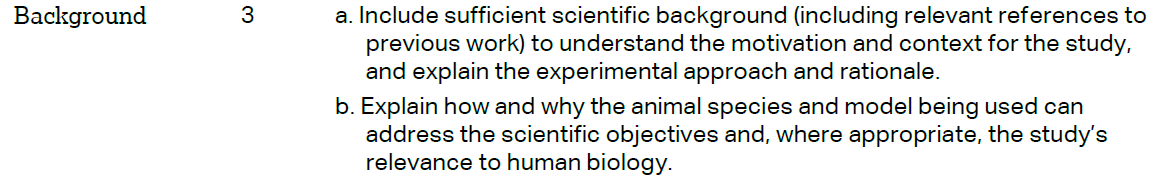 | | | Paragraphs 1-3  Paragraphs 2-3 |  |
| 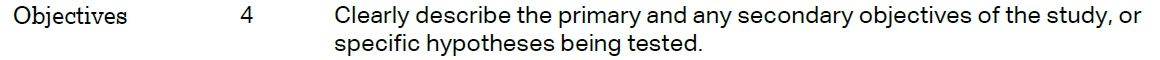 | | | Paragraph 3 |  |
| METHODS | | |  |  |
| 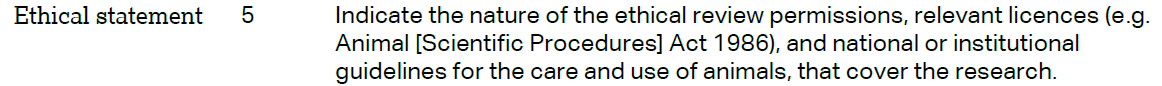 | | | Paragraph 4 |  |
| 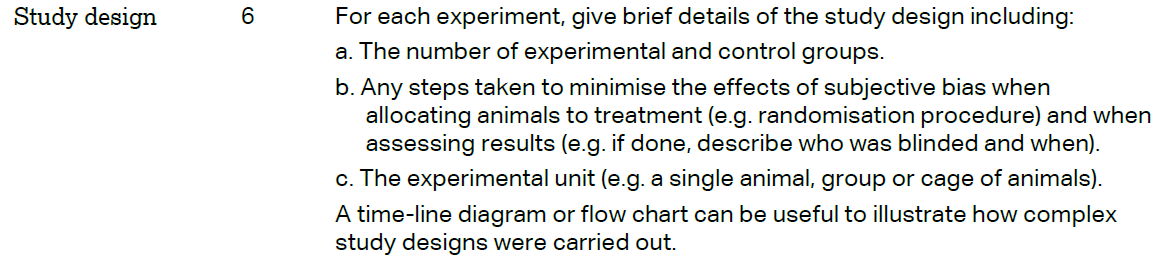 | | | Paragraph 5 and Figures 2-6, 9 |  |
| 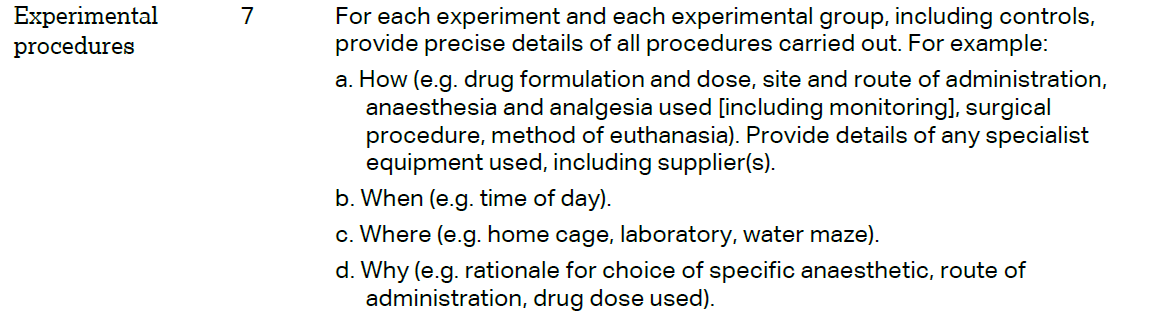 | | | Paragraphs 1-9 |  |
| 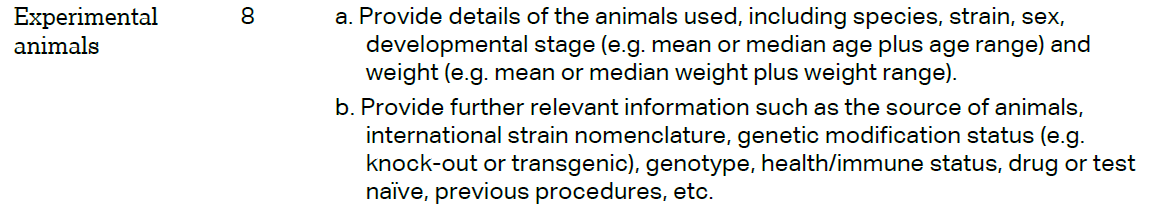 | | | Paragraph 1 |  |

The ARRIVE guidelines. Originally published in *PLoS Biology*, June 2010^1^

| 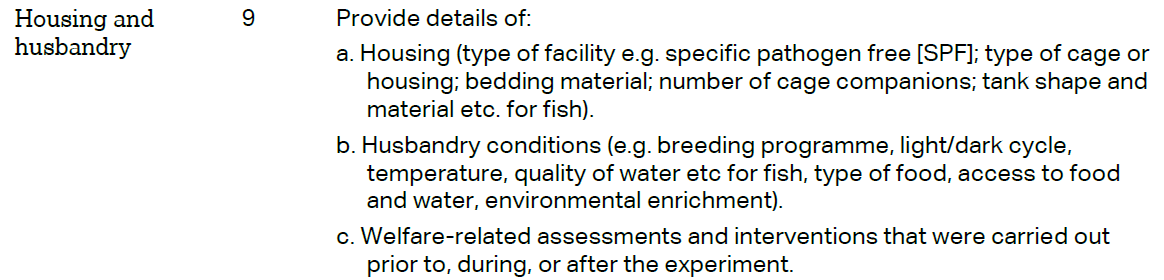 | Paragraph 4 | |
| --- | --- | --- |
| 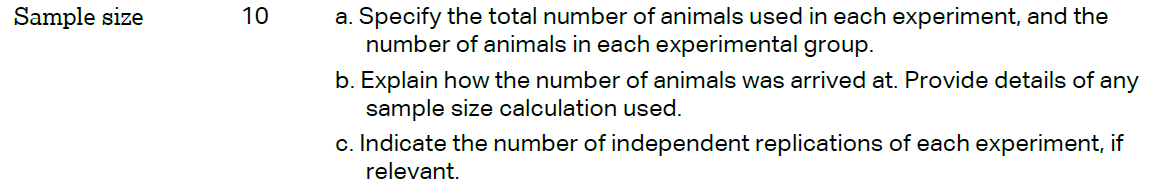 | Paragraph 5 and Figures 2-6, 9 | |
| 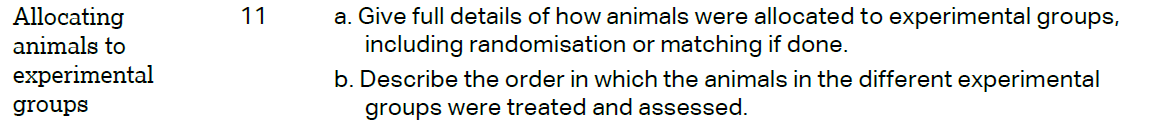 | Not applicable | |
| 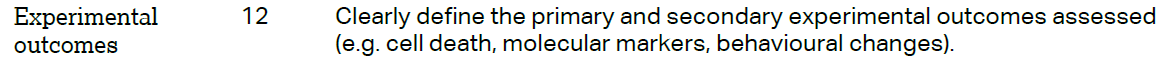 | Paragraphs 2-3, 5-7 and 9 | |
| 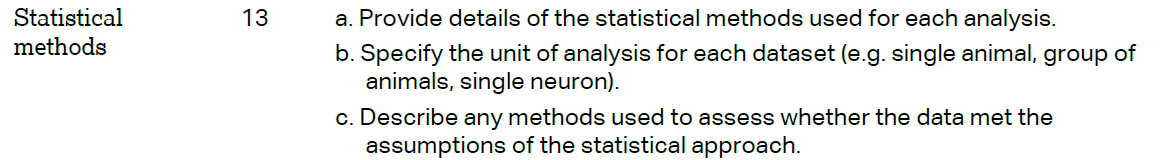 | Paragraphs 8-9 and Figures 6 and 9 | |
| RESULTS |  | |
| 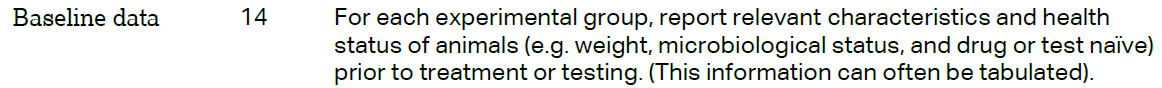 | Methods/ Paragraph 1 and Figure 6 | |
| 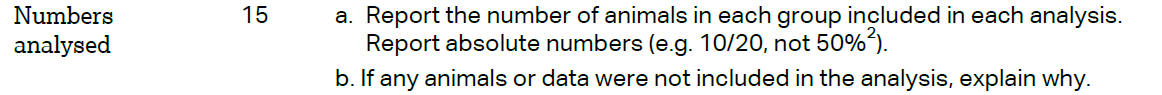 | Paragraph 11, Figure 6 and Methods/ Paragraph 9 | |
| 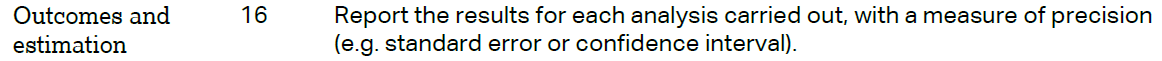 | Methods/ Paragraph 8 and Figures 6,9 | |
| 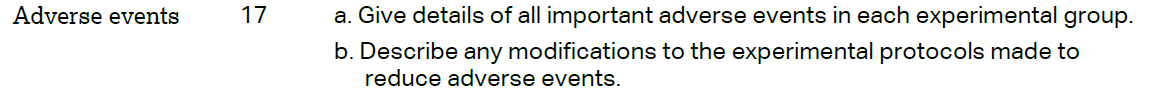 | Paragraphs 10 and 12 | |
| DISCUSSION |  | |
| 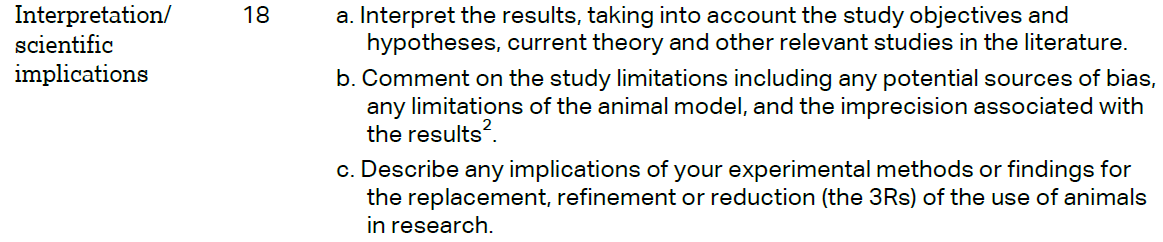 | Paragraphs 3-8  Paragraphs 1, 2  Paragraph 8 | |
| 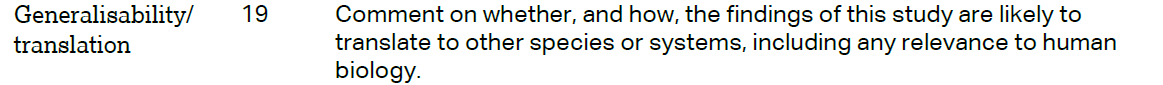 | Paragraphs 6-8 and Results/ Paragraph 8 | |
| 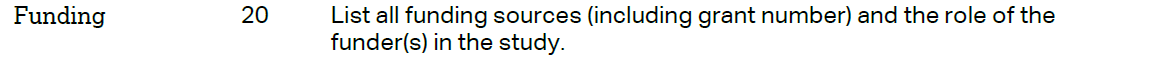 | | Cover Letter |


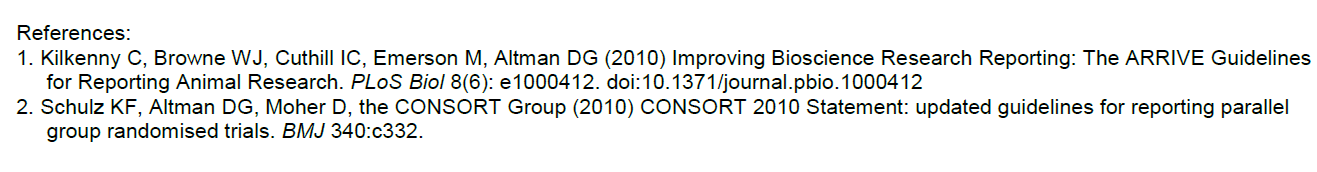

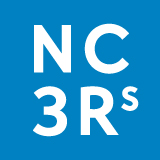

Supplement: S1 ARRIVE Guidelines Checklist — (DOCX) [file pone.0125522.s001.docx]
