# Supplementary figures and images for: Diverse Phenotypes and Specific Transcription Patterns in Twenty Mouse Lines with Ablated LincRNAs
Source: PLoS One. 2015 Apr 24;10(4):e0125522. doi: 10.1371/journal.pone.0125522 (PMC4409293; doi:10.1371/journal.pone.0125522)

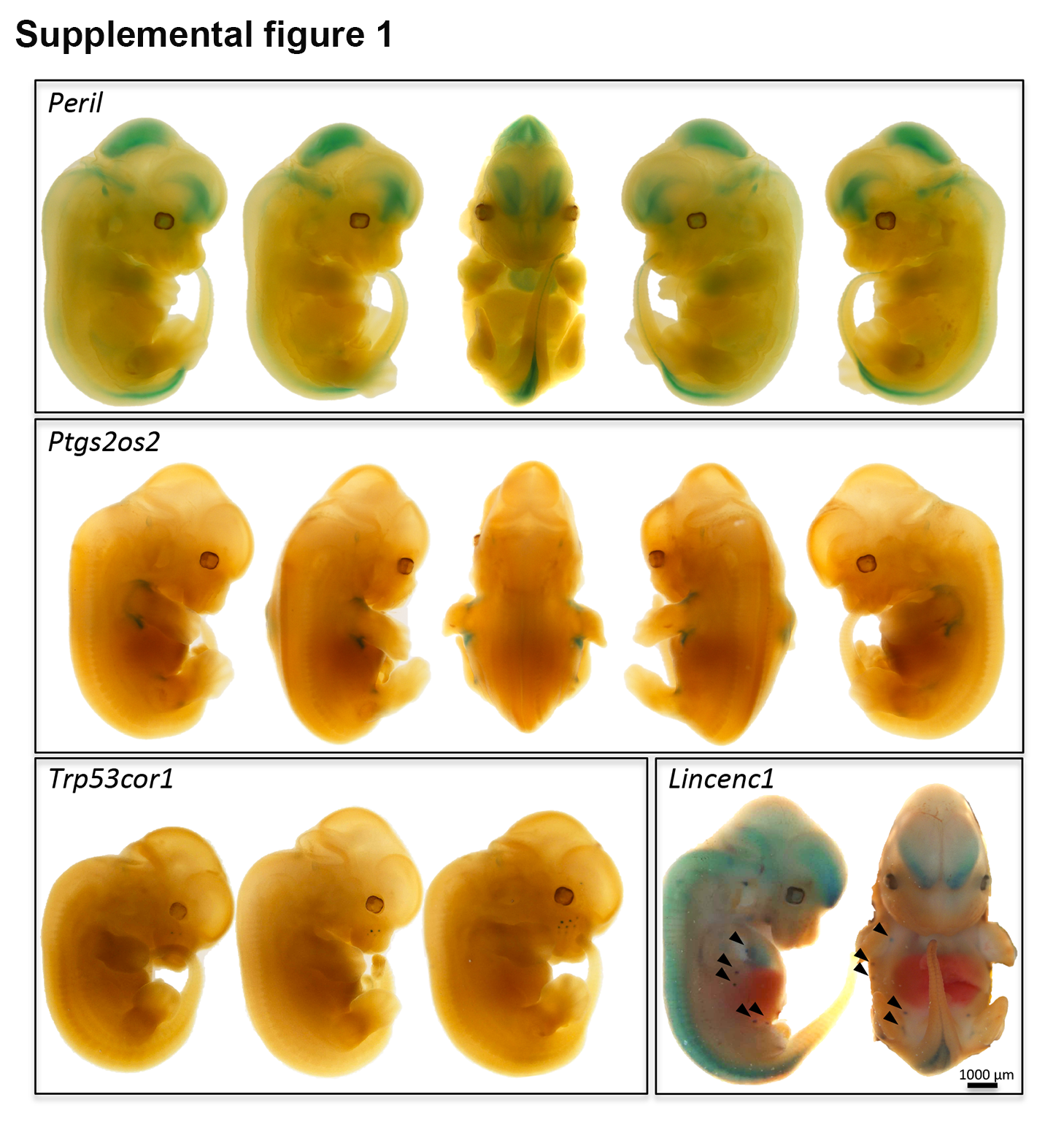

Supplement: S1 Fig — (A) LacZ reporter profiling for Peril shows a specific neuronal expression pattern as well as strong expression in the heart and posterior tail region. (B) Ptgs2os2 lacZ reporter expression is restricted to the base of developing forelimbs and hindlimbs. (C) Trp53cor1 lacZ reporter expression is specific to the developing whisker placode in the nasal process. E12.5 embryos collected from the same litter capture the progression of whisker placode development over a short period of time. (D) Forelimbs and hindlimbs were removed in the Lincenc1 +⁄− embryos to reveal mammary bud expression (arrowheads). Ventral view of E12.5 Lincenc1 +⁄− embryo: lacZ expression is detected in five pairs of mammary buds. (TIF) [file pone.0125522.s002.tif]

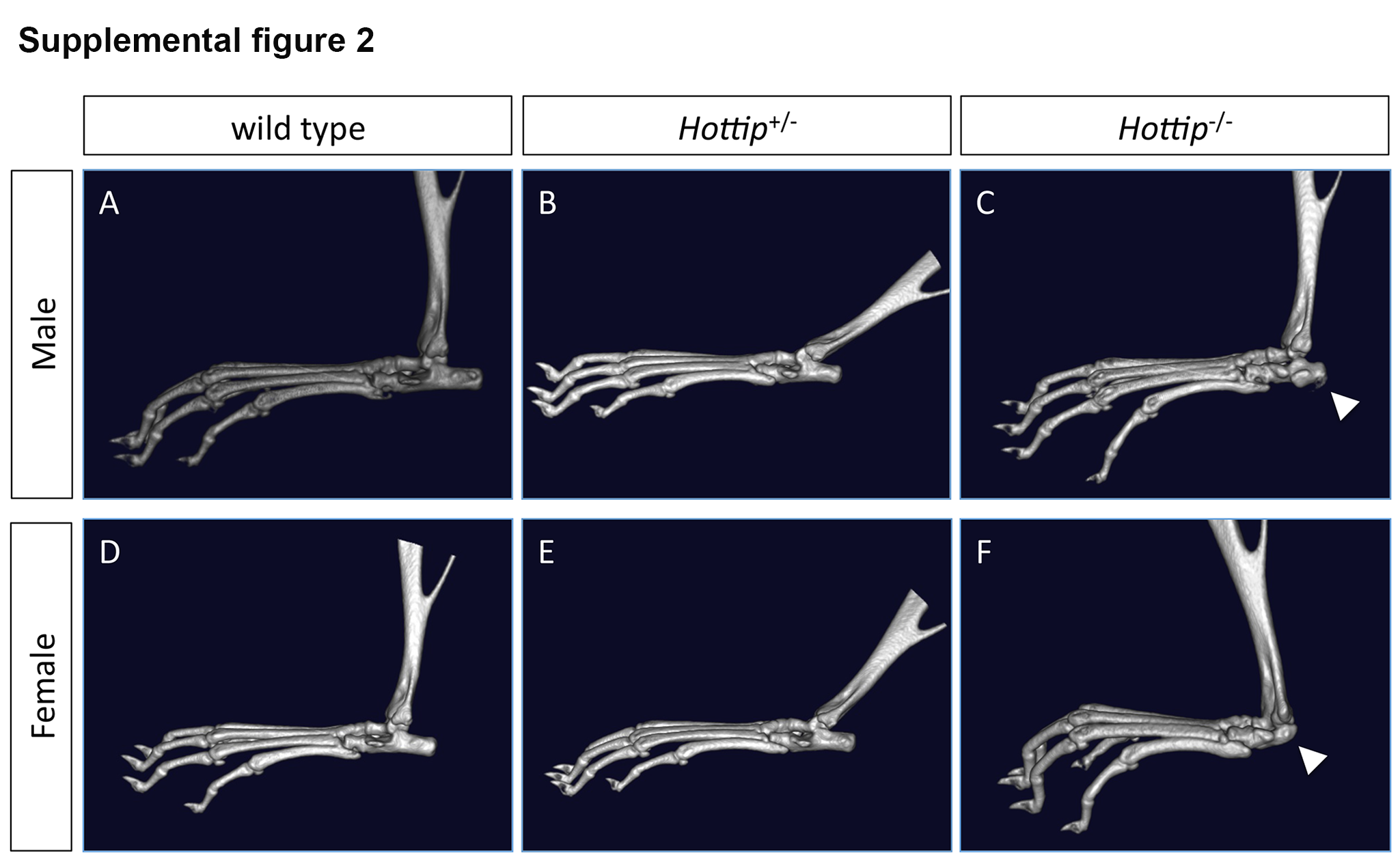

Supplement: S2 Fig — In addition to a skeletal muscle phenotype in the hindlimb, Hottip −⁄− mice also display a skeletal bone abnormality visualized by 3D microCT. Both male and female (C and F) Hottip −⁄− mice have shortened calcanea (arrows) in comparison to (A and D) WT and (B and E) Hottip +/− littermate controls. (TIF) [file pone.0125522.s003.tif]
